# Diagnostic techniques in deflagration and detonation studies

**DOI:** 10.1186/s13065-015-0128-x

**Published:** 2015-09-28

**Authors:** William G. Proud, David M. Williamson, John E. Field, Stephen M. Walley

**Affiliations:** 1 Surface Microstructure and Fracture Group, Cavendish Laboratory, University of Cambridge, J.J. Thomson Avenue, Cambridge, CB3 0HE United Kingdom; 2 Institute of Shock Physics, Imperial College London, Blackett Laboratory, Prince Consort Road, London, SW7 2AZ United Kingdom

**Keywords:** High-speed, Quantitative, Experimental, Diagnostic, Characterisation

## Abstract

**Abstract:**

Advances in experimental, high-speed techniques can be used to explore the processes occurring within energetic materials. This review describes techniques used to study a wide range of processes: hot-spot formation, ignition thresholds, deflagration, sensitivity and finally the detonation process. As this is a wide field the focus will be on small-scale experiments and quantitative studies. It is important that such studies are linked to predictive models, which inform the experimental design process. The stimuli range includes, thermal ignition, drop-weight, Hopkinson Bar and Plate Impact studies. Studies made with inert simulants are also included as these are important in differentiating between reactive response and purely mechanical behaviour.

**Graphical abstract:**

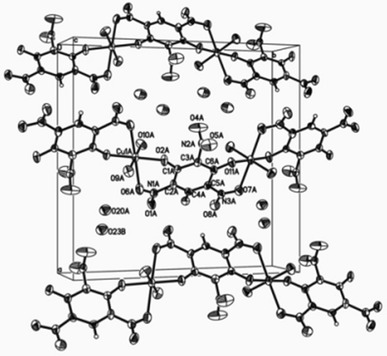

## Background

Energetic materials give high energy-release rates resulting in phase transformation, rapid temperature rise, producing mechanical and other work. These dramatic changes occur on a sub-millisecond to sub-nanosecond timescales, making them inaccessible to many standard techniques. Initially, studies focused on post-reaction effects e.g., crater size in metal blocks, or impulse given to ballistic pendulums. These studies should not be underestimated, however, the interpretation applied to results was quite broad, so the resulting theories reflect the limitations in understanding the high-speed processes.

Increased use of explosives resulted in a number of explosive accidents and poisonings [1], so legislation was introduced with the aims of reducing risk to property and people. This resulted in a series of qualification and classification protocols to address common hazard scenarios [2]. They also produced a series of reference scales to allow the comparison of data from different sources. While this ranking is often useful, given the nature of the test fundamental processes may be masked by the presence of complicating factors.

With an increasing importance of environmental issues, safety and handling and reliability, numerical modeling and prediction has become a major area offering the promise of wide applicability, a shorter timescale and lower cost than a large experimental series. However, accurate prediction requires accurate knowledge of fundamental behaviour coupled with well-designed experiments. This increases interest in physical understanding of the reaction processes.

Modern data capture techniques; which give increased capture rates and sensitivity have allowed increased understanding and the availability of nanosecond time-resolved data is increasingly common [e.g., in reference 3]. As a result small-scale tests and sensitive techniques have been developed to populate and validate predictive models.

This paper presents a number of techniques to give an overview of some important material parameters and processes. This is not an exhaustive account nor are the techniques applicable to all cases; however, they show the need for well-controlled stimuli, giving clear results. In some cases the understanding of previously unknown or secondary processes has led to another cycle of development.

## High strain rate regimes

Table 1 shows the range of strain rates addressed in this review, along with the corresponding techniques. A review of techniques that have been applied over this range of strain-rates has been published by the authors elsewhere [4]. This present review, however, the techniques applied to energetic materials; drop weight studies which are generally used to determine ignition levels, Hopkinson bar studies in relation to impact scenarios, while plate impact produces stresses associated with detonation. Increasing strain rate and the sudden delivery of energy to a sample allows processes with high activation energies to be accessed. Processes that operate on long time scales such as thermal diffusion do not have time to occur.

**Table 1:** High strain-rate regimes, the associated equipment and stimulus duration

Strain rate	Equipment	Stimulus duration	Comment
10^−6^–10^−2^	Instron	100 s of seconds	Quasi-static loading
10^+2^–10^+3^	Drop weight	10 s milliseconds	Generally used to determine impact ignition thresholds
10^+2^–10^+3^	Hopkinson bars	100 s microseconds	Compression, tension and torsion loading. Extensively used for PBX formulations. Constitutive models
10^+4^–10^+5^	Miniature Hopkinson bar	10 s microseconds	For fine grain materials or single crystals. Generally metals
10^+3^–10^+6^	Taylor impact	10 s microseconds	Sometimes used for metal jacketed energetic samples
10^+5^–10^+8^	Plate impact	Microseconds	Pressures and durations similar to that of gap tests. Laser driven flier plates have sub-microsecond duration high-intensity shocks

In general yield and fracture stresses increase with strain rate [5] in particular at strain rates >10^3^ s^−1^, which is also the regime where the effect of sample inertia becomes significant. Ultimately under shock-loading the sample response changes from one of stress equilibrium to of a wave-controlled process where the material is severely compressed in the principal loading direction but does not have the time to move laterally.

## Techniques and diagnostics

### Quasi-static techniques

Quasi-static loading forms the bedrock of all material characterization. Properties traditionally measured in this way are density, heat capacity, melting point, ignition point, molecular and crystal structure. Advances in X-ray techniques such as tomography have allowed the internal structure of powders and crystals to be determined in great detail [6]. Atomic force microscopy permits chemical composition, hardness and topology to be measured at a nanometer level [7]. Environmental scanning electron microscopy allows insight into variations on a sub-crystal length scale. The analysis of data from many areas rely on these properties which often need to be experimentally determined as they are not easy to predict e.g. the density of a polymer-bonded explosive.

**Fig. 1:**
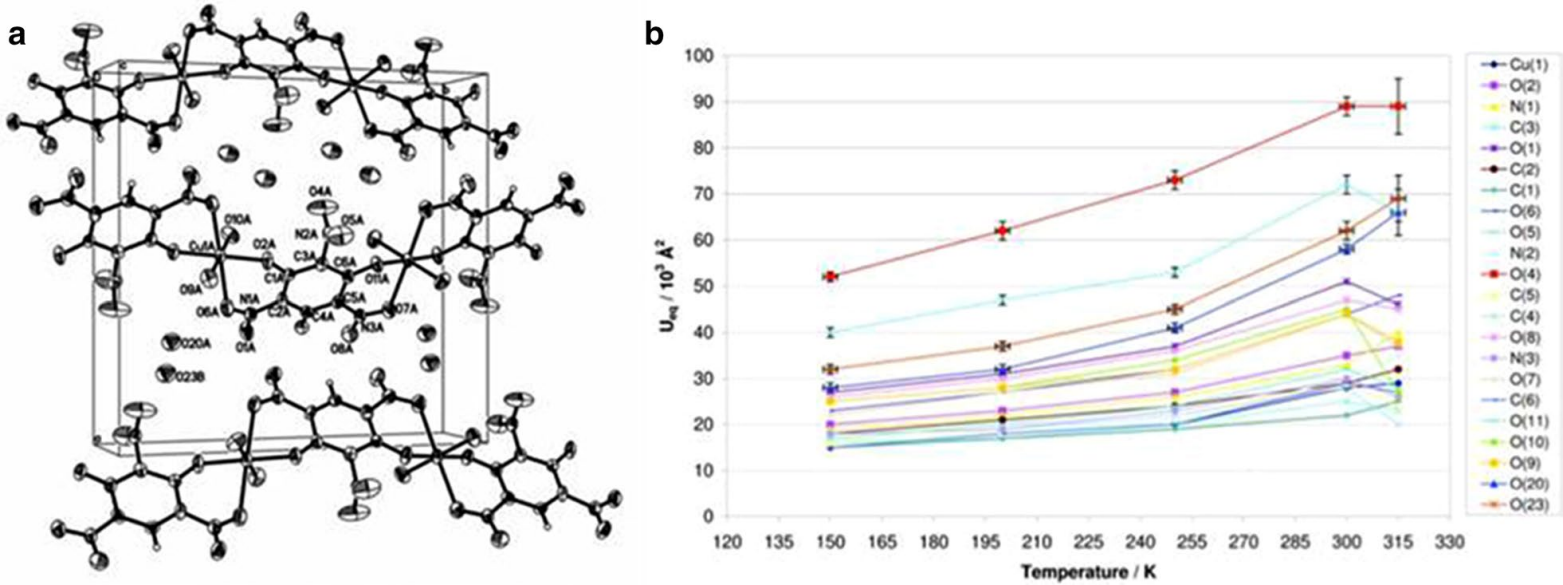
**a** Crystal structure **b** Crystal parameters of copper (II) styphnate tetrahydrate in terms of atomic displacement factor (*U*_*eq*_) [8]

X-ray diffraction is used for structural determination over a range of temperatures. The crystal structure is associated with material sensitivity as this determines the ease of slip along crystallographic planes. Temperature controlled X-ray diffraction is useful in linking the sensitivity of molecular and crystal-cell properties, determining the heat capacity and phase transitions such as the movement of water molecules within the crystal structure. Figure 1a shows the structure of copper (II) styphnate tetrahydrate; Fig. 1b the temperature variation of the crystal parameters. In Fig. 1b it can be seen that all the atomic displacement (*U*_*eq*_) factors for the components of the molecules increase in a similar fashion, until ~300 K, indicating a regular expansion and excitation of the crystal structure. Above 300 K the long-range order breaks down, when parts of the structure display displacements increasing at a greater rate than the other compoments, eventually, decomposition occurs [8]. A more in-depth analysis shows initially the water of crystallization moves, rapidly followed by bulk breakdown. This shows the fundamental limitation of this material, which cannot be resolved by simple manufacturing changes.

Polymer bonded explosives have been subject to much research into understanding the effect of fracture [9–12]. If fractures move through the polymer binder they will be important in weakening the material but are not so obviously related to increasing material sensitivity. However, cracks that run through the energetic filler particles, opening up new interfaces in energetic crystals which can then rub against each other are a more obvious route to increased sensitivity. Data from moiré interferometry and other image correlation techniques, combined with micromechanical models, show the importance of the strain rate behaviour of the polymer binder. In general polymers become harder with decreasing temperature or with an increase in strain rate [12]. The adhesion between crystal and binder is also very important in strain localization process and the production of critical hot-spots [11].

### Deflagration to detonation studies

Localisation of energy into inhomogeneities within an energetic material produces hot spots [13, 14] from which reaction can build and spread. Several mechanisms such as void collapse have been identified as important. These mechanisms act simultaneously, sometimes it is difficult to identify which is dominant, however, once sufficient critical hot spots are produced, deflagration will start to spread. The resulting deflagration has been studied in a variety of ways using high-speed photography.

**Fig. 2:**
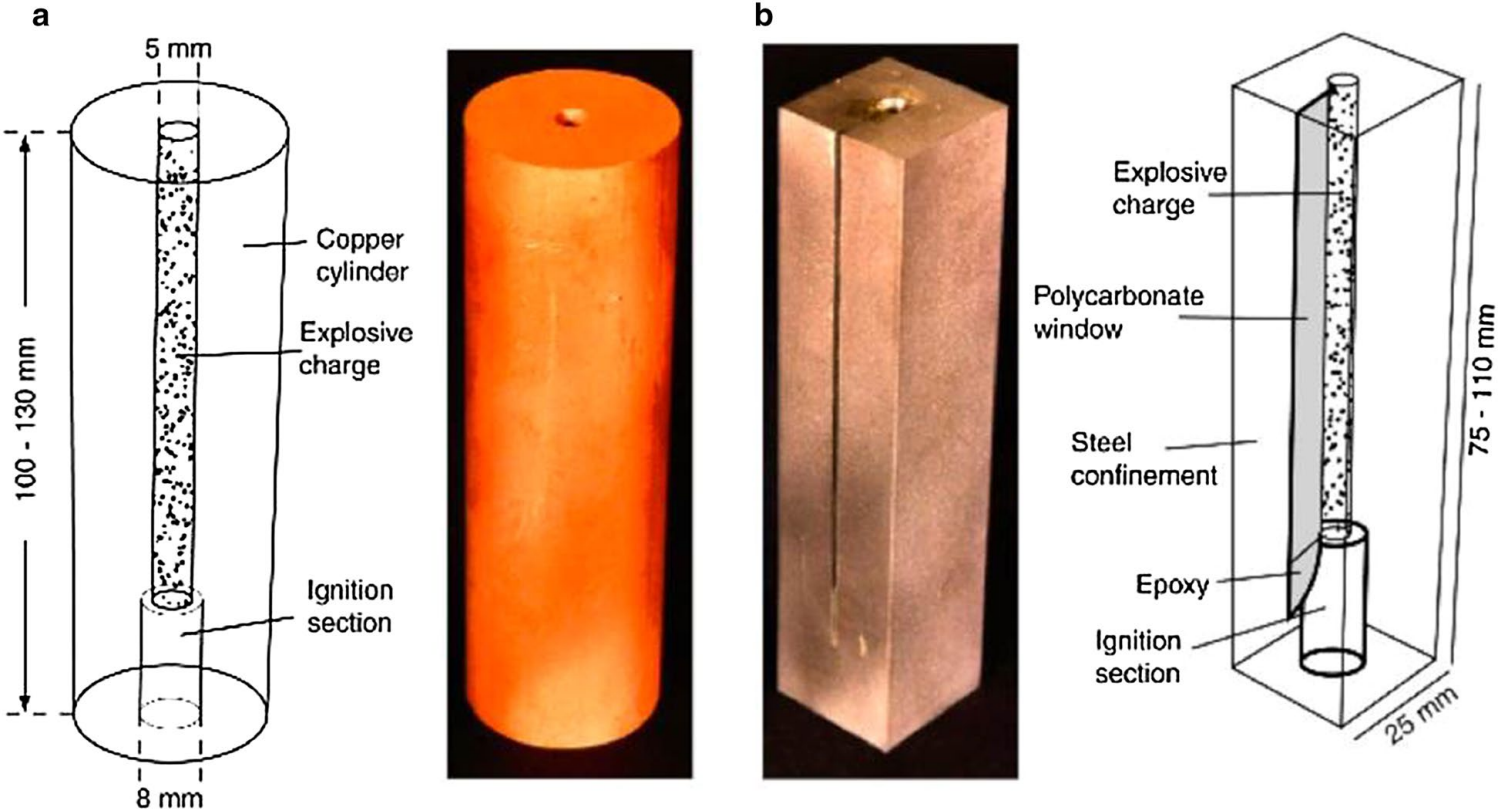
Cylinders used in **a** post-experimental **b** photographic studies of DDT [15]

The simplest experiments use regular geometrical shapes such as the thick-walled cylinders shown in Fig. 2. These cylinders are 70–100 mm long and have a central bore 5 mm diameter, filled with the energetic materials of interest. Initial analysis of what had occurred was performed by post-experimental examination. Experiments carried out with copper, steel and brass cylinders showed copper gave the clearest record, while brass was too brittle and steel although providing some record did not distort in the ductile manner of copper so providing a less clear picture.

Plastics cylinders, in general do not provide the required level of confinement [15] for a deflagration to detonation (DDT) event. When a photographic record was required [15] the confinement shown in Fig. 2b, developed by Luebcke [16], was used, based on previous designs by Korotkov [17] and Griffiths and Groocock [18]. The confinements consisted of sections of square steel bar, a 5 mm channel drilled along the centre, and a 1 mm wide polycarbonate window laid into the steel. The window allows imaging while being sufficiently thin that the level of confinement is not overly compromised.

Using a hydraulic press, the explosive charges were incrementally pressed to 75 % of theoretical maximum density (TMD); care was taken to ensure that each pressing increment never exceeded more than half of the diameter of the column to reduce density variation along the column. Charges with densities of ~50 % TMD were also incrementally pressed but required more modest force to be applied in this case static weights were placed on top of the pressing rod.

In many cases, a thin piece of copper foil was placed between the ignition section and the explosive charge. This prevented light from the burning of the ignition charge from being transmitted to the optical fibres used to trigger the experimental diagnostics.

Pyrotechnic ignition was used, the mixture being 80 % potassium dichromate and 20 % boron as developed by Dickson [19]. The pyrotechnic was added after the main charge had been pressed into the column. The pyrotechnic was tamped in order to ensure as few gas pockets as possible and was ignited using a nichrome wire heated by an electrical current. This system has the advantage of producing few gaseous products and the burn temperature is far higher than the ignition temperature of the energetic materials used.

To keep a pressure seal on the column a small aluminium cone was placed around the electrical wires and pushed into the confinement to reduce rearward venting of the charge. As a result pressure generated during the early stages of reaction could be sustained.

**Fig. 3:**
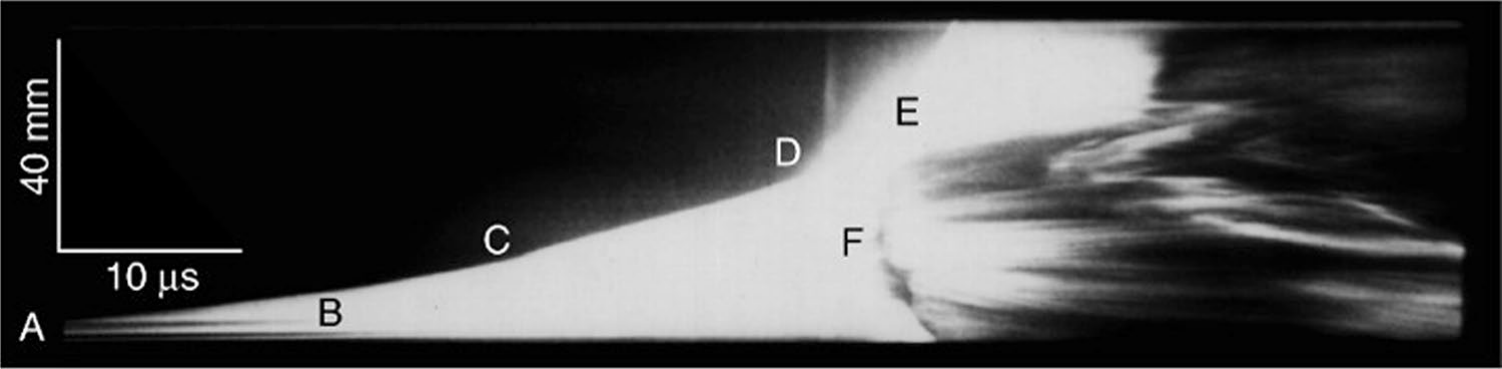
DDT event in ultrafine PETN [21]

The type of data recorded is shown in Fig. 3. A full description can be found in Gifford [20–22]. The streak image shows convective burning B, the step from deflagration to detonation D and the movement of the waves including a retonation wave F. High-speed thermocouples [21] were sometimes added and allowed the condition at points along the column to be measured.

### Drop weight studies

The drop-weight is a standard device used for sensitivity studies that allows millisecond long, low-level pressure pulses to be applied to small samples. If high-speed photography, photodiodes and stress transducers are used as diagnostics this gives a powerful system for qualitative and quantitative understanding.

**Fig. 4:**
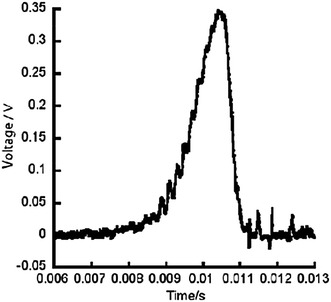
Typical output from a drop-weight stress transducer used in these studies [4]

The standard and simple analysis of a drop weight experiment assumes the weight is a rigid body to which Newton’s laws of motion apply. This analysis is used in determining the dynamic calibration of drop weight force transducer, directly relating the force to the deceleration of the weight and, via integration the drop-weight position. A typical transducer signal is shown in Fig. 4.

During impact a drop weight machine shows stress wave oscillations comparable in magnitude to the mechanical resistance of the specimen as the impact excites the weight below its resonance frequency [23]. Elastic waves reverberate to bring the weight to rest and in many cases cause the weight to rebound. Recent research [24–27] shows that high quality data can be obtained from such machines.

**Fig. 5:**
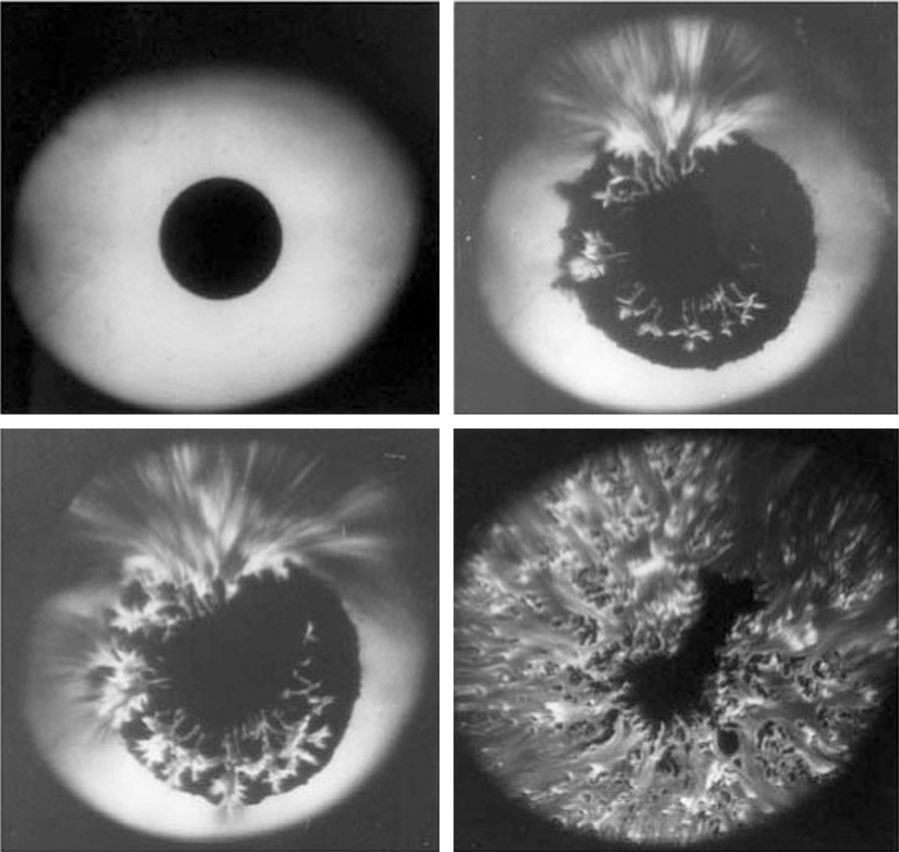
The ignition under impact of a thermite composition in a transparent anvil drop-weight [31]

One modification, which has proved invaluable in studies of explosive ignition, is to use glass or sapphire anvils [14, 28–31]. This gives a light-path in the direction of drop-weight impact and allows high-speed photography. A ‘classic’ high-speed photographic sequence using this modification is shown in Fig. 5.

### Second harmonic generation

In some cases the test material may possess properties that allow specific techniques to be applied. One of the optical properties of HMX was used to probe its ignition under drop-weight impact [32]. Second harmonic generation (SHG) was used to study the β-δ phase transition in HMX in the late 1990s [33–36]. A second harmonic is produced when radiation interacts with molecular crystals of appropriate symmetry; sometimes this is called ‘frequency doubling’. β-HMX has a “chair” configuration and is centrosymmetric: forbidden from generating second harmonics. However, δ-HMX, has a “boat” configuration, lacks a centre of inversion and efficiently generates second harmonics. To allow these second-order processes to be observed the incident radiation has to be intense, laser illumination is required. A Nd:YAG, (YAG = yttrium aluminum garnet) laser, operating at 1064 nm, was used allowing the detection of the second harmonic at 532 nm (green), in the region of many cameras peak sensitivity. The second harmonic generation is near instantaneous process and so a 9 ns laser pulse, can probe for δ-HMX during impact.

**Fig. 6:**
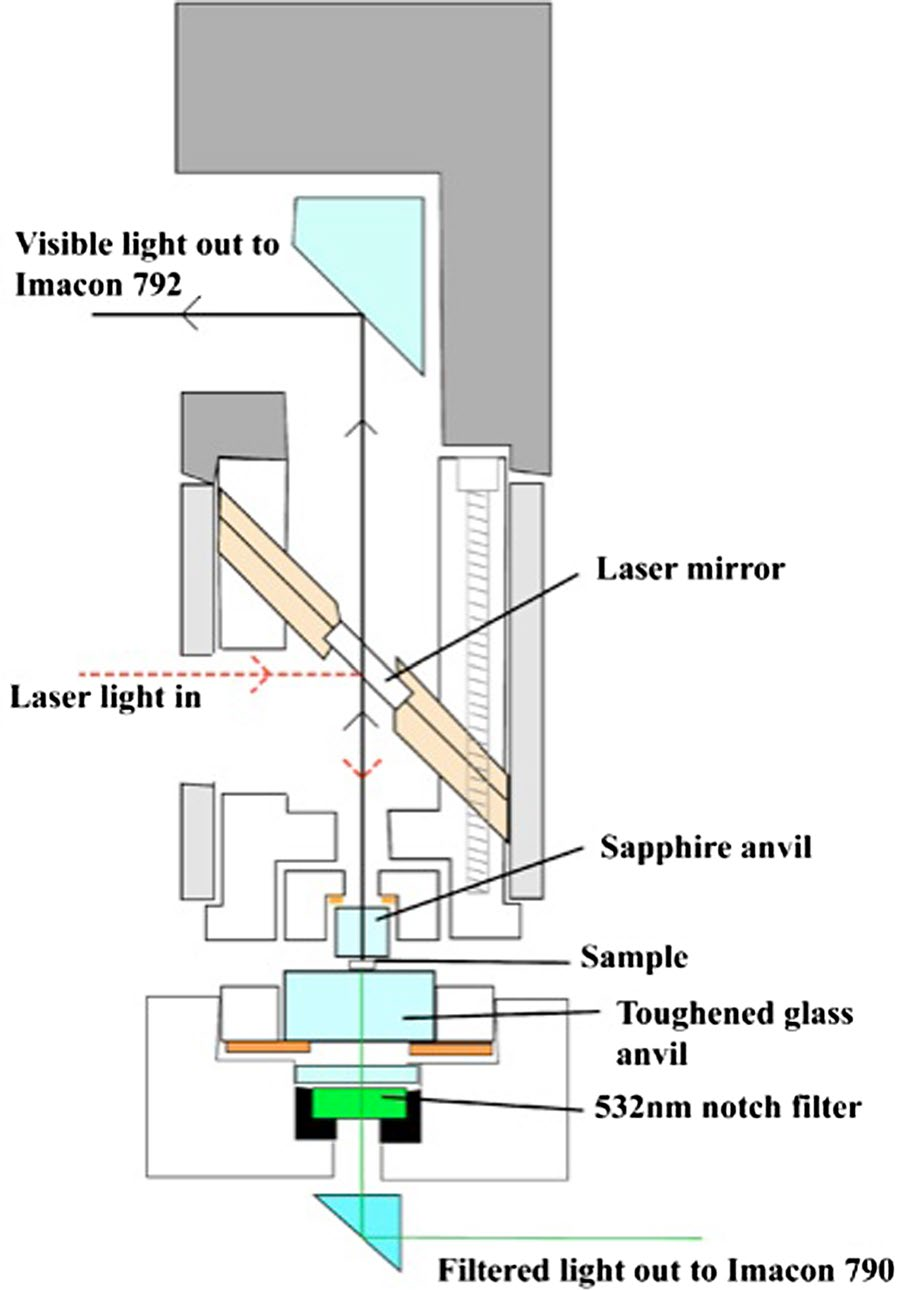
Drop weight modified to detect SHG in HMX crystals during impact [32]

A drop weight with transparent anvils was used to stimulate reaction in a pressed pellet of β-HMX, as shown in Fig. 6. Separate light paths allow laser light to illuminate the sample while visible from reaction or SHG can be captured by Imacon 790 and 792 cameras. A notch filter in front of one camera isolated the SHG light from any δ-HMX produced, while the other camera followed the visible light.

A 5.5 kg mass dropping from 39 cm gave an impact velocity of 2.75 m s^−1^ and would reliably ignite a 50 mg β-HMX sample. The form of the stress history was a half sinusoid of peak pressure 10^7^ Pa and 10^−4^ s duration. A light gate was used to trigger the diagnostics just before impact, while a photodiode monitored ignition. The cameras were set such that the visible light camera started to record just before the laser while the first frame on the filtered camera coincided with the laser pulse.

Using β-HMX pellets with one surface converted to δ-HMX tested the sensitivity of the system to δ-HMX. SHG was visible independent of the δ phase being on the top or the bottom of 1 mm thick pellet with excellent spatial resolution.

**Fig. 7:**
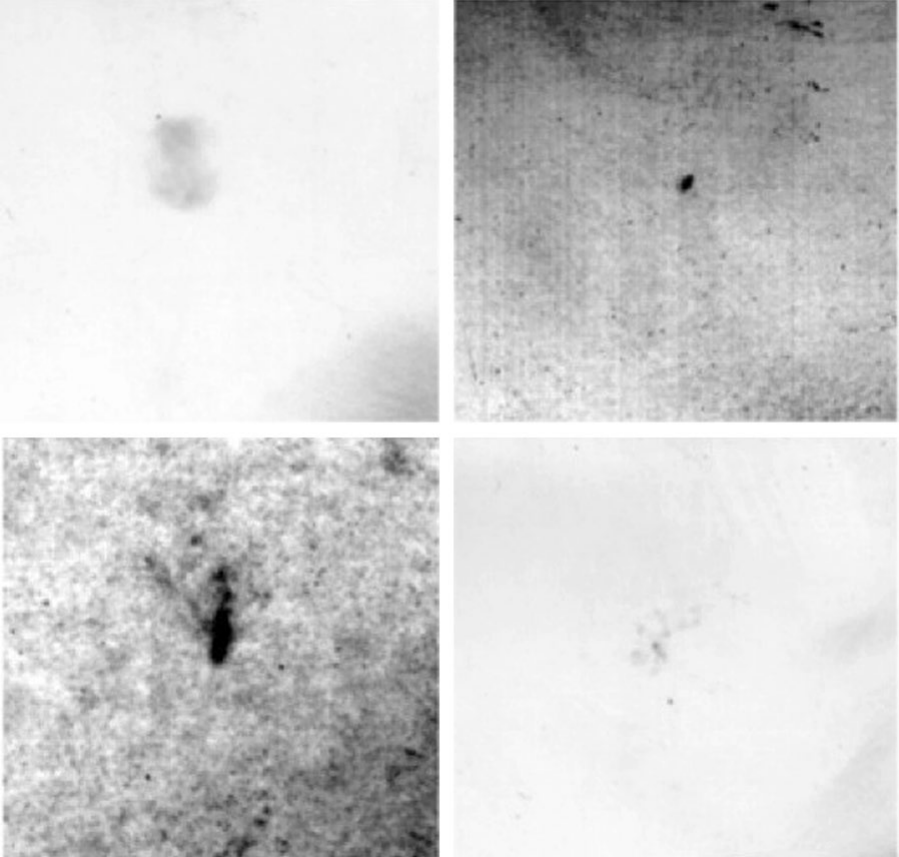
Four images, each from a different experiment showing SHG. All images taken less than 13 μs in advance of ignition. All images have enhanced and reversed contrast;* black* indicates the presence of δ-HMX. Field of view of is 10 mm wide [32]

Selected images showing SHG are presented in Fig. 7. The images show second harmonic in narrow regions and δ-HMX was never observed any earlier than 13 μs before ignition. This implies that δ production occurs late in the reaction sequence.

Field [37] saw localized heating and attributed this to shear band formation. It appears that the heating associated with impact is sufficient to cause small, localized patches of δ-HMX (~1 mm across) in the region of shear bands. After ignition, the heating caused by reaction produces larger patches, ~3 mm across, before the sample was completely consumed.

### Digital speckle photography

**Fig. 8:**
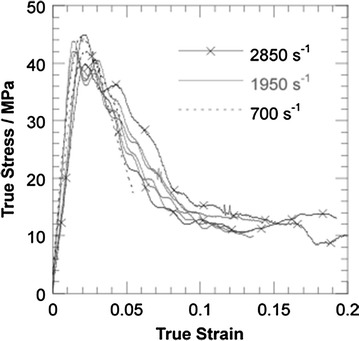
Stress-strain curves showing results for a PBS. Two repeat experiments are shown at three different strain rates [40]

Hopkinson bars are used to probe rates of 10^2^–10^4^ s^−1^, an important region in strain rate characterization associated with dramatic increases in the dynamic strength. Many polymers are rate sensitive becoming brittle in this strain-rate regime. In general the output of Hopkinson Bar studies takes the form of stress–strain curves (Fig. 8), a description of the technique can be found in [4]. The standard data analysis for this loading system is based on conservation of sample volume i.e., no fractures as well as homogenous material flow.

Micro-mechanical models are being developed and require validation data that includes the motion of the particles and binder as well as the development of fractures. In this case the technique of digital speckle photography can be used. This is a method that compares the movement of random patterns to produce strain maps. These patterns may be naturally present, as in the case of large grain-size PBXs, or can be painted onto the surface.

**Fig. 9:**
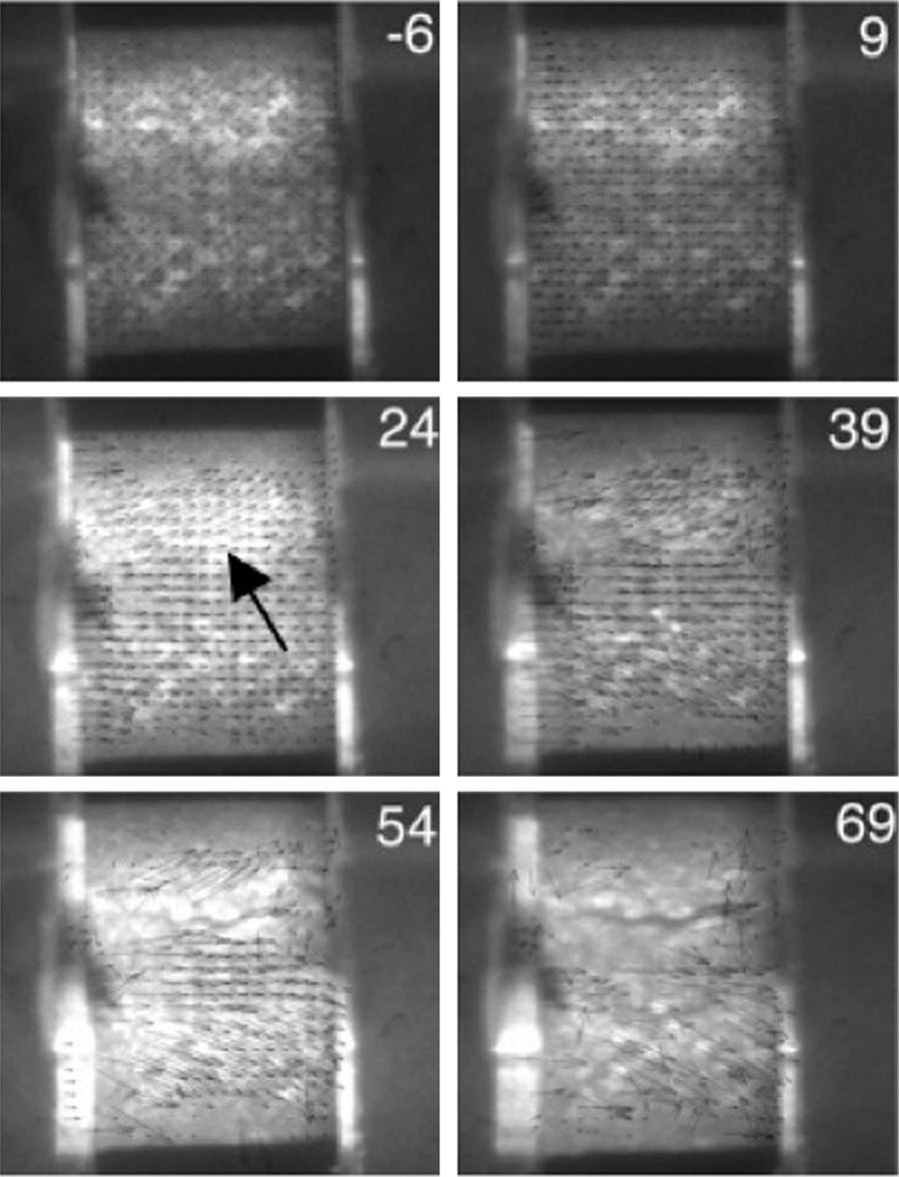
Displacement evolution using image correlation in a sample of PBS under Hopkinson Bar Loading. The number indicates microseconds after compression starts, the* black arrow* the onset of fracture

In the study presented here an inert PBX simulant, a polymer-bonded sugar (PBS), was sprayed with colloidal silver ‘*dag*’ to produce the speckle pattern. High-speed photographs were taken during the loading from the Hopkinson bar and analyzed using software described in [38–40]. The resulting strain maps, for a sample deformed at a strain rate of 1420 s^−1^ are shown in Fig. 9.

Specimen diameter was independently measured using the shadow cast during loading by illuminating a line wider than the sample diameter with a line laser and measuring the intensity of the light arriving at a photodiode. As the sample expands the amount of light seen at the photodiode decreases.

**Fig. 10:**
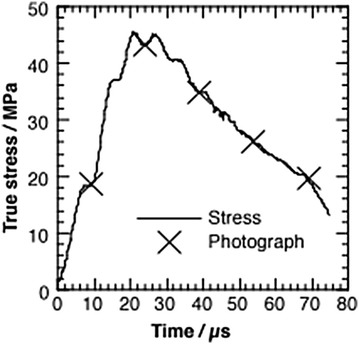
Stress-time plot for the PBS sample in Fig. 9. The crosses correspond to the images in Fig. 9

The evolution of displacement on the surface of a typical specimen is given in Fig. 9. The arrow in the frame at 24 µs shows the onset of strain localization. Surface cracks are visible in the next frame. Comparison with Fig. 10 shows localization corresponded to the peak stress. In this case localization, cracking etc. precede strain softening.

**Fig. 11:**
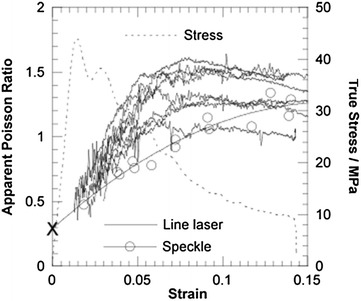
Comparison of apparent Poisson’s ratios from line laser and speckle for 3 specimens. Also shown are the small strain Poisson’s ratio (X) and a typical stress–strain curve [40]

**Fig. 12:**
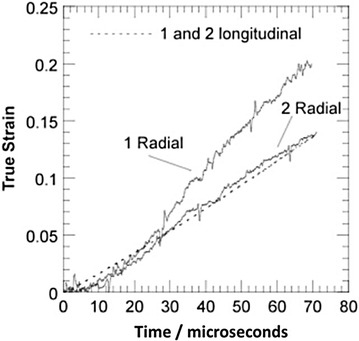
Comparison of longitudinal strains from the standard Hopkinson bar analysis to radial strains from the line laser for two specimens

Speckle data were used to calculate strain fields in both the longitudinal and radial directions to produce an apparent Poisson’s ratio for different strains, Fig. 11. The small strain Poisson’s ratio, calculated from ultrasonic measurements, is ~ 0.30 [41]. This data indicates that the use of single values relating strain within samples is not supported by dynamic data, the situation is more complex. This is shown in Fig. 12 where the line laser output was used to measure the radial strain for two samples, deformed at the same strain rate. They display the same longitudinal strain throughout but develop different radial strains after 30 microseconds.

**Fig. 13:**
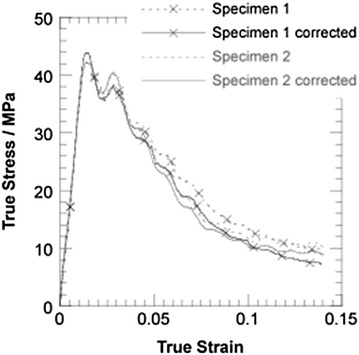
Comparison of stress–strain curves calculated assuming volume conservation in the specimen, and those curves which use the apparent Poisson’s ratio (‘corrected’). Only a small difference is seen

This shows early onset of fracture: the area of the *specimen* is not the load bearing area. The true stress in the material may be better represented by assuming volume conservation in the material not volume conservation of the specimen. To obtain measurements in material volume conservation would require significant development in, for example, dynamic tomography. However, it is encouraging to note that, as the key features of the mechanical behaviour for these materials occur at small strains, where the difference between the stress–strain curve assuming volume conservation, and the observation using the line laser measurement is small. Overall the variation in the results between assuming volume conservation and using the data from the laser system is less than the difference seen between specimens, Fig. 13.

### Digital speckle radiography

In the last three examples, shock waves will be considered. In this case mechanical behaviour of an inert simulant, followed by a study of energetic sensitivity and finally detonation studies used to understand processed occurring within the detonation front.

Digital Speckle Radiography (DSR) is the x-ray equivalent to Digital Speckle Photography (DSP) [42] relying on the cross-correlation of image subsections. DSR makes use of X-ray images combined with the placing of a lightly populated, <30 %, layer of X-ray opaque particles within the sample. The benefit of DSR over DSP is that it allows for the measurement of the internal deformations. Short duration of flash X-rays, 70 ns pulse width, are ideal for ‘freezing’ fast processes such as those found in high-speed impact [43].

Here the DSR technique is applied to experiments simulating the effects of setback and set-forward on the explosive fill of a munition. These effects result from the high inertial forces exerted during gun launch (setback) and impact/penetration (set forward) [44].

An inert polymer bonded simulant of sugar in a hydroxy-terminated polybutadiene (HTPB) matrix, represented the energetic filling. The speckle field was created by seeding lead particles on the central plane perpendicular to X-ray axis, parallel to the impact axis. The lead particles were 500 μm in diameter and the coverage was ~20 % by area.

The X-ray system was a Scandiflash 150 keV unit producing a 70 ns X-ray pulse. Medical grade intensifier screens and film were used to capture the images on film, which was subsequently digitally scanned.

In these experiments most targets were loaded by single shock wave [45]. However, an experiment was performed in which the sample was doubly shocked. Double shock scenarios have important implications for explosive initiation [46].

**Fig. 14:**
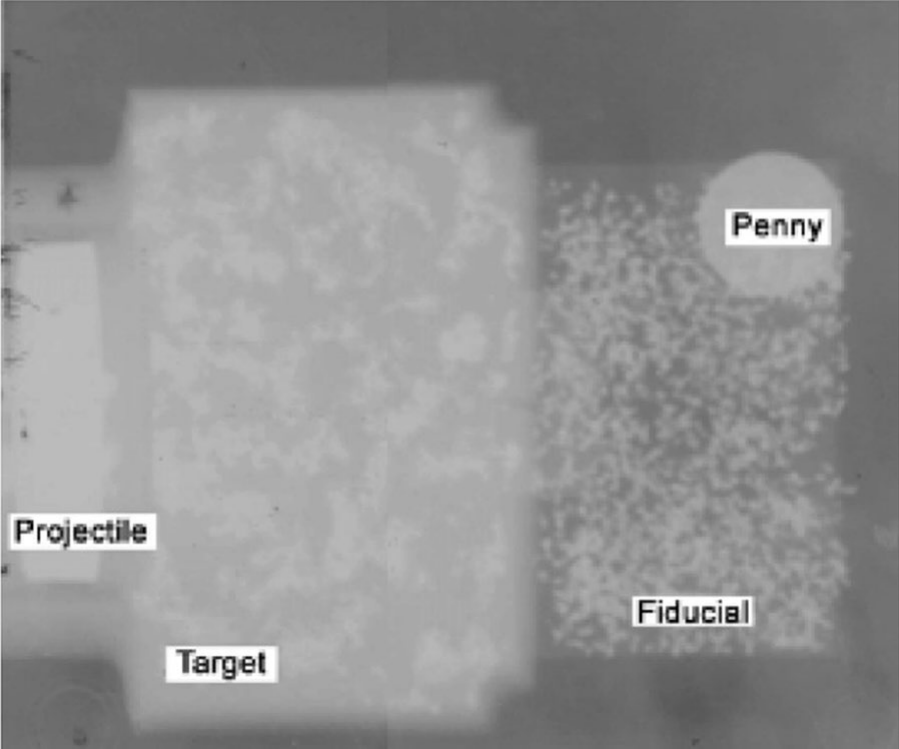
Flash X-ray of impact experiment. The UK 1 pence piece acts as a indicator of the field of view. The projectile traveling left to right and has struck the target, the fiducial markers do not move on the timescale of the experiment. [44]

Figure 14 shows a typical X-ray image obtained when the projectile has just struck the target. The fiducially speckle field is used to remove any rigid body motions introduced during the scanning process. Also visible is the silhouette of a UK 1 pence piece, which acts as a scale bar (diameter 20.3 mm).

**Fig. 15:**
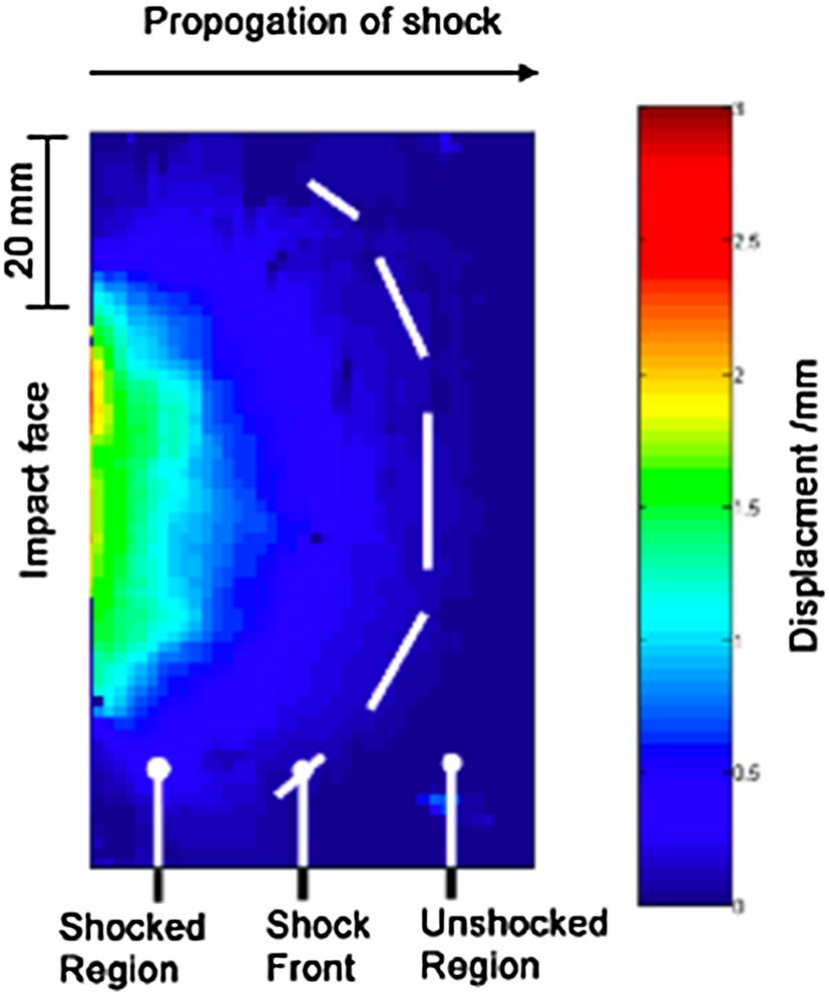
u-component data, displacement along the line of impact, from plate impact experiment

**Fig. 16:**
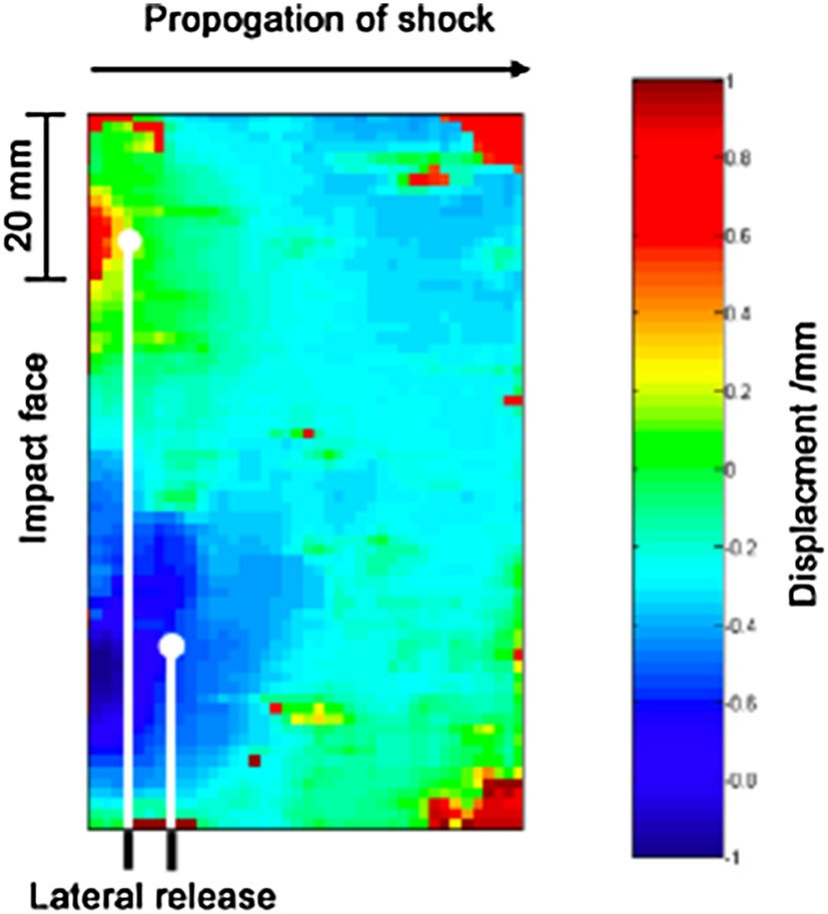
v-component data, displacement orthogonal to the impact direction, from plate impact experiment

Here the u-axis was parallel to the projectile axis, and the v-axis is perpendicular. Figure 15 shows the u-component of displacement within the target area, the shock wave is clearly visible. The curvature of the shock front is due to lateral release of material from the shocked to an unshocked state. Lateral release waves originate from the edges of the impacting projectile. Information about the lateral release is most easily seen in the v-component data, Fig. 16.

Using displacement curves reported in [44], and knowledge of the time delays between the experiments, the shock wave velocity was 1.6 km s^−1^ for impact at 300 m s^−1^ and 2.4 km s^−1^ for impact at 600 m s^−1^. These data is also used to determine the strain associated with the shock: 4.0 ± 0.1 % for 300 m s^−1^ impact and 7.3 ± 0.1 % for 600 m s^−1^.

**Fig. 17:**
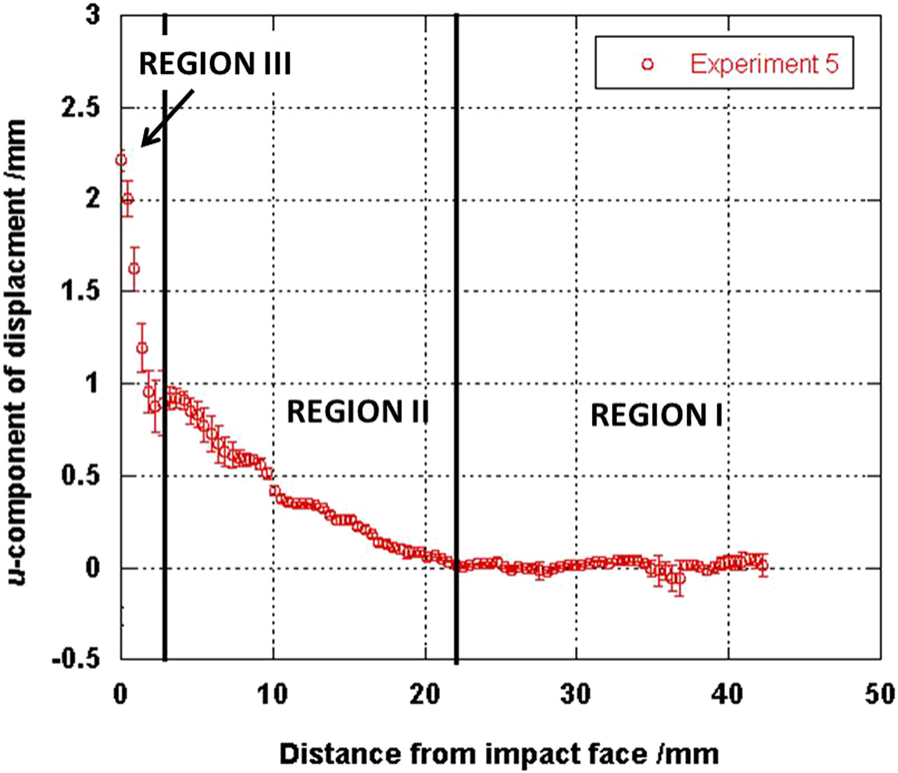
Displacement measured in PBS material subject to a stepped shock wave. The unshocked region I has no displacement, the initial low shock region II shows displacement indicating compression, region III involving the high shock level has a rapid change in displacement indicated significant compression

A stepped shock wave was sent through the target using a composite impactor. The resulting displacement is shown in Fig. 17. Region I is unshocked material, while region II is material subject to the first shock level, having a strain of 5.5 ± 0.1 %. Region III is material subject to the second, higher shock state; the strain in this region is 60 ± 5 %. This data has been used both to populate and validate material models. This example also shows the advantage of using inert simulants to measure behavior that would have been masked by reaction in the corresponding energetic system.

### Small scale gap test

In the gap test the shock sensitivity of a material is determined by placed it in contact with a barrier and exposed to a shock pulse produced on the other side of the barrier, the ‘gap’, by an explosive charge. As the shock from the charge moves through the barrier it dissipates: the thicker the barrier the lower the shock felt by the sample. Repeating the experiment with different barrier thicknesses allows a 50:50 or go: no-go threshold to be established.

**Fig. 18:**
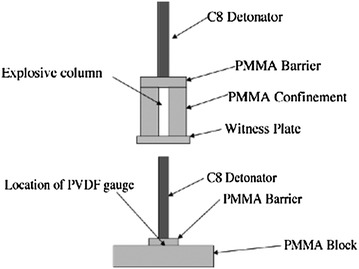
Experimental arrangements. *Top*, for gap test, *Bottom*, for PVDF gauge study

In this study small samples of energetic [47] were pressed into a central bore, 5 mm diameter, in PMMA cylinders 25 mm long, 25 mm in diameter. Incremental pressing with an increment height of 0.5 mm gave a very homogeneous column. Column densities from 60 to 90 % TMD were studied. The gap test arrangement is shown in Fig. 18. A PMMA gap with a C8 detonator on top is placed on the column. At the lower end of the arrangement is a brass witness plate. All contacting surfaces were coated in a thin layer of silicone grease in order to allow good acoustic transmission between the layers.

The camera was triggered when light was detected in a fibre optic fitted near the end of the detonator.

**Fig. 19:**
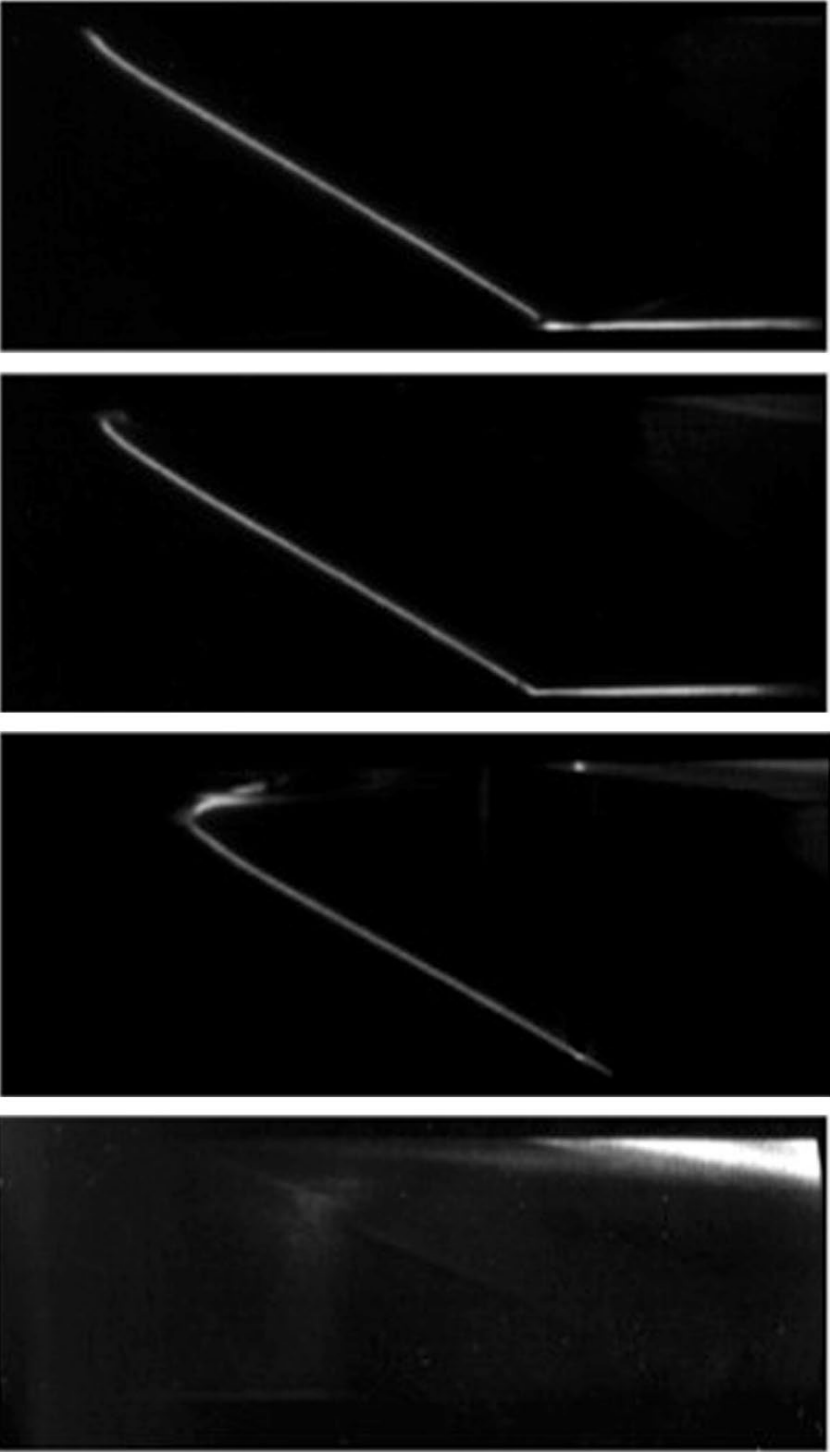
Streak records of gap tests on fine-grained PETN 90 % TMD. Gap thickness; top—3.53 mm, second—3.63 mm, third—3.67 mm, bottom—3.71 mm

Streak records from a series of experiments on 90 % TMD ultrafine grain size PETN are presented in Fig. 19. The field of view is 30 mm and the top of the image corresponds to the bottom of the gap. The gaps used were 3.51, 3.63, 3.67 and 3.71 mm thick. In the top image the detonation is prompt and slightly overdriven. The detonation velocity settled down within 5 mm of column length. The second image shows slight hooking due to the detonation starting a short distance down the column, indicating a longer initiation time. With the 3.67 mm gap the hooking is severe, detonation breaks out ~8 mm down the column. Finally with a 3.71 mm gap there is no detonation. The brass witness plate indicated a detonation pressure in all cases except for the 3.71 mm gap where only a small dent was found indicating the more modest pressure associated with deflagration. A similar series of results was obtained for conventional grain size material where the gap thickness for failure was shown to be 5.54 mm of PMMA.

**Fig. 20:**
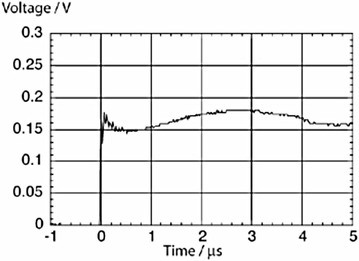
PVDF Output from a gauge separated by 5.54 mm PMMA gap from a C8 detonator

To calibrate the system Dynasen PVDF gauges [48] were placed between a gap plate and a block of PMMA. A typical PVDF gauge output from these experiments is shown in Fig. 20, the output voltage can be converted to stress. For a 5.54 mm PMMA gap gave a stress level of 2.1 GPa while a gap of 3.67 mm gave 4.1 GPa. This indicates that the fine-grained PETN would not detonate when exposed to approximately twice the shock pulse magnitude of that required to initiate conventional PETN in this particular experimental arrangement.

### The cylinder test

**Fig. 21:**
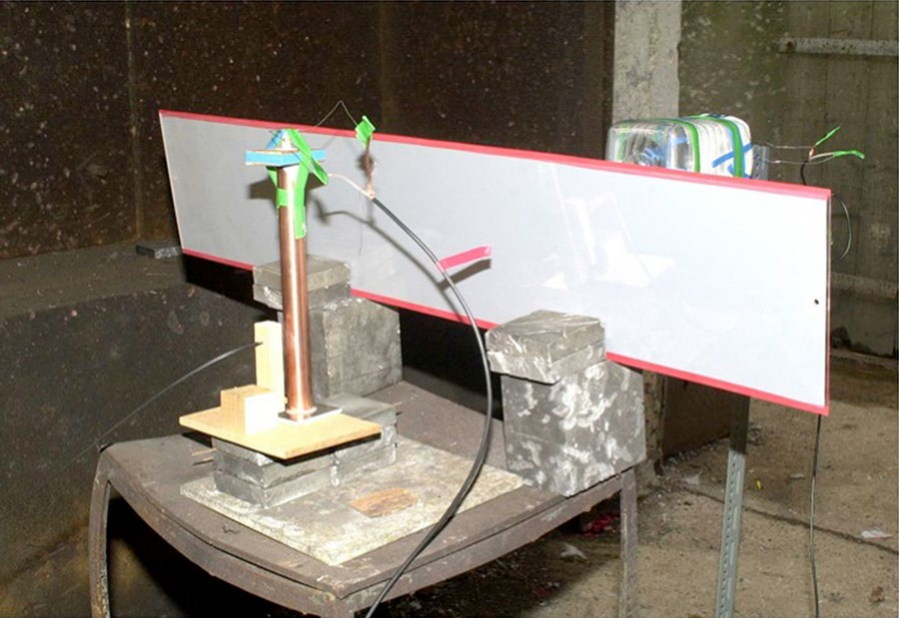
The cylinder test arrangement

Cylinder tests are used to measure detonation performance and populate numerical models using a very simple arrangement, a metal tube, usually copper, filled with explosive. The material is detonated and the deformation of the tube is measured and this is related to the detonation pressure and velocity, the arrangement is shown in Fig. 21. High-speed recording systems, such as streak photography, allow the rate of expansion of the cylinder expansion to be measured.

However, if the aim is to track changes within the detonation front a more time-resolved technique may be required. In this the diagnostics were supplemented by the use of a velocity interferometer (VISAR) [49] developed by Barker et al. in the late 60’s and early 70’s [49].

VISAR uses the Doppler shift associated with reflecting light from an accelerating surface. The reflected light is captured and split into two beams, one of which passes through a glass cylinder known as an “etalon”. The glass slows the light and when it emerges it is delayed in time with respect to the other beam, which has passed through air. If acceleration has occurred combining the two beams will produce interference, a beat frequency, which allows the surface velocity to be determined. The time resolution of a VISAR system is of the order 2 ns.

A streak camera recorded the radial expansion of the cylinder while VISAR measured the velocity history of the outer surface of the cylinder [50]. Copper cylinder expansion tests were carried out on nitromethane/aluminium (NM/Al) compositions containing between 20, to 60 % weight aluminium particles. The NM was Analar grade and the aluminium particles were spheres with a mean diameter of 10.5 μm. The copper cylinders were 304 mm long, with an inner diameter of 25.4 mm and a wall thickness 2.6 mm, sealed at the bottom. A small booster pellet initiated the charges.

**Fig. 22:**
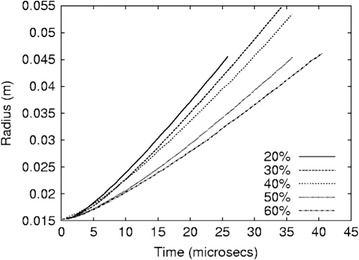
Radius expansion from streak images. Varying mass fraction of Al in NM

The expansion was backlit by an argon flash bomb and a diffusing screen was recorded with a Cordin-132 streak camera writing at 6.0 mm/μs. Expansion velocity decreased as the particle loading density increased with a marked change was between 40 and 50 % loading as shown in Fig. 22. The initial expansion phase from these data is limited by the resolution of the camera and film.

**Fig. 23:**
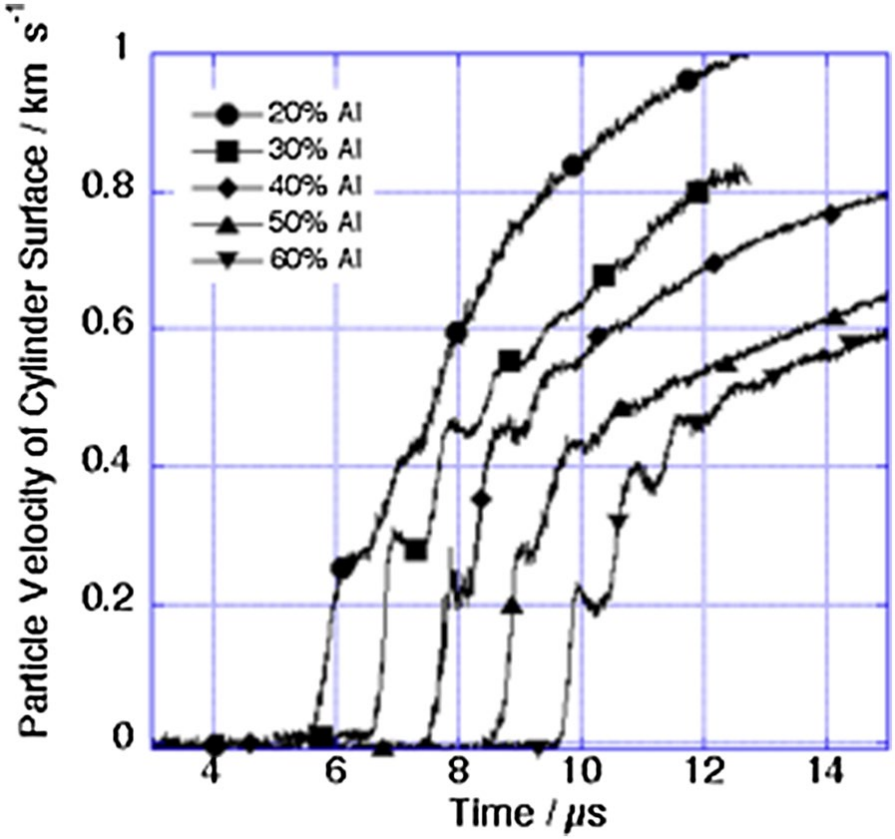
Velocity histories captured by VISAR for various Al loadings

**Fig. 24:**
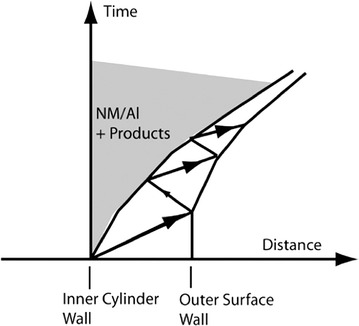
Schematic of wave reflections in the cylinder walls

**Fig. 25:**
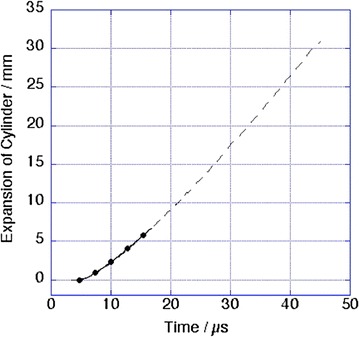
Combined VISAR (*solid*) and streak (*dashed*) records for 60 % Al loading

Analysis of the VISAR traces gave the velocity histories shown in Fig. 23, in which the traces are offset for clarity. The initial acceleration takes the form of a series of steps, due to the reflection of waves within the copper cylinder, these can be explained using Fig. 24. The detonation of the NM/Al mix sends a shock wave into the inner cylinder wall. This reflects at the outer wall as a release front, which travels back through the tube. When the release reaches the inner wall it is reflected as second shock due to the pressure from the detonation products. This reverberation process gives rise to the velocity steps seen in the VISAR trace until a final velocity is reached. Ultimately the expansion produces thinning in the copper cylinder walls and they shred into fragments. These velocity steps are not seen in the record as they occur in the first 2 µs, where the displacements are very small. Overall the streak camera system is excellent for large displacements it cannot capture the detail of the early acceleration. The displacements given by the streak and VISAR traces are combined in Fig. 25 and show close agreement.

## Conclusions

These examples show a wide range of techniques applied to energetic systems, ranging from low to high velocity impact but they are far from exhaustive. However, it is possible to draw some general conclusions. Using simple experimental geometries is useful as it allows data to be extracted with limited recourse to complex analysis. Attention to sample preparation, particularly the use of small increments to minimize sample density variation, gives a marked reduction in error bars allowing processes to be clearly observed. In energetic materials, it has been known for over 50 years that ignition and initiation are multi-variable processes, so experimental design is of paramount importance.

In some cases like HMX, the specific material properties allow techniques such as second harmonic generation to be used.

While many of the techniques listed can involve complex equipment, some advances, like the use of line lasers to monitor material expansion require modest resources.

Most importantly it is essential to have a clear idea of the desired output from the study. Where the requirement for the study is a legal one, such a qualification test for placing of a material on the market or transporting it, variation in procedure may invalidate the results. However, if it is a study to evaluate material properties, experiments can be adapted to lead to new data, which can reveal the complex and fundamental behaviour of reactive materials.
